# Integrating and validating automated digital imaging analysis of estrogen receptor immunohistochemistry in a fully digital workflow for clinical use

**DOI:** 10.1016/j.jpi.2022.100122

**Published:** 2022-06-30

**Authors:** Saba Shafi, David A. Kellough, Giovanni Lujan, Swati Satturwar, Anil V. Parwani, Zaibo Li

**Affiliations:** Department of Pathology, Wexner Medical Center at The Ohio State University, 410 W. 10th Ave, Columbus, OH 43210, USA

**Keywords:** ER, Digital image analysis, Visiopharm, Breast cancer, Clinical

## Abstract

**Background:**

The Visiopharm automated estrogen receptor (ER) digital imaging analysis (DIA) algorithm assesses digitized ER immunohistochemistry (IHC) by segmenting tumor nuclei and detecting stained nuclei automatically. We aimed to integrate and validate this algorithm in a digital pathology workflow for clinical use.

**Design:**

The study cohort consisted of a serial collection of 97 invasive breast carcinoma specimens including 73 biopsies and 24 resections. ER IHC slides were scanned into Philips Image Management System (IMS) during our routine digital workflow and digital images were directly streamed into Visiopharm platform and analyzed using automated ER algorithm to obtain the positively stained tumor nuclei and staining intensity. ER DIA scores were compared with pathologists’ manual scores.

**Results:**

The overall concordance between pathologists’ reads and DIA reads was excellent (91/97, 93.8%). Pearson Correlation Coefficient of the percentage of ER positive nuclei between the original reads and VIS reads was 0.72. Six cases (3 ER-negative and 3 ER-positive) had discordant results. All 3 false negative cases had very weak ER staining and no more than 10% positivity. The causes for false positive DIA were mainly pre-analytic/pre-imaging and included intermixed benign glands in tumor area, ductal carcinoma in-situ (DCIS) components, and tissue folding.

**Conclusions:**

Automated ER DIA demonstrates excellent concordance with pathologists’ scores and accurately discriminates ER positive from negative cases. Furthermore, integrating automated biomarker DIA into a busy clinical digital workflow is feasible and may save time and labor for pathologists.

## Introduction

Evaluating the expression of estrogen receptor (ER) is a standard practice for breast carcinoma since it harbors both prognostic and predictive value.^1^, ^2^, ^3^ ER status should be determined on all primary and recurrent breast carcinomas based on the American Society of Clinical Oncologists (ASCO)/College of American Pathologists (CAP) guidelines.^4^ ER expression is usually evaluated manually by estimating positively stained tumor cells via viewing immunohistochemistry (IHC)-stained slides under light microscope, but inter- and intra-observer variability occurs frequently.^5^, ^6^, ^7^, ^8^, ^9^, ^10^, ^11^, ^12^, ^13^

The widespread implementation of whole slide imaging (WSI) and the rapid development of deep learning (DL)-based algorithms have generated enormous interest in artificial intelligence (AI)-driven computational pathology technologies, including automated quantitative digital imaging analysis (DIA) of biomarkers. While manual interpretation of IHC is a subjective and time-consuming process, automated DIA offers the possibility of producing rapid, uniform results with improved precision.^14^ Indeed, excellent correlation has been demonstrated between manual and DIA scoring of ER IHCs in breast carcinoma and higher reproducibility has achieved by using DIA than manual scoring.^15^, ^16^, ^17^, ^18^, ^19^ Some studies have examined algorithms that require input and training by pathologists,^20^^,^^21^ while others have used unsupervised algorithms without any training or prior data.^22^ However, almost all these studies required separate slide scanning, WSI uploading to DIA platform, manual selection of region of interest (ROI), causing additional workload and delayed results, which may not be suitable for a busy pathology practice. In the current study, we validated an automated ER DIA coupled with the preexisting WSIs which have already been scanned during our routine digital workflow.

## Materials and methods

### Patients and specimens

After institutional review board approval at The Ohio State University, a pathology archive database search was performed for a period of 1.5 years from August 2020 to January 2021 to retrieve 97 surgical pathology cases with a diagnosis of invasive breast carcinoma and with a quantitative ER result. The cases represent a serial collection of breast carcinomas received at our hospital between August 2020 and January 2021.

### Estrogen receptor immunohistochemistry

ER protein was assessed on formalin-fixed paraffin embedded (FFPE) (ischemic time < 1 h and fixation time between 6 and 72 h) whole tissue sections by immunohistochemistry (IHC). An automated deparaffinization step was followed by cell conditioning and then rinsing and incubation with the pre-diluted anti-ER antibody clone SP1 (Spring Bioscience) at 37 °C. Staining was performed using Leica/Bond polymer detection system on a Leica/Bond auto-stainer. The slides were counterstained, then rinsed and cover slipped.

### Pathologists’ scoring

ER IHC was manually scored as a percentage of positive tumor cell nuclei and staining intensity according to ASCO/CAP guidelines. The percentage of positive tumor cell nuclei was categorized as <1% (negative), 1–10% (low positive), or >10% (positive). The overall staining intensity was categorized as weak, moderate, or strong. ER IHC results were signed out by board-certified breast pathologists (original reads). In addition, 2 pathologists independently scored ER IHCs by manual semi-quantification during this study.

### Image acquisition, management, and automated digital image analysis (DIA)

ER IHC slides were scanned into whole slide images (WSI) using the Philips scanners and stored in Philips Image Management System (IMS) consisting of Philips IntelliSite Pathology Solution 3.2 systems (IMS software version 3.2.1, Ultra-Fast Scanner [UFS] serial No. FMT0145 with software version 1.8, and Philips display PP27 QHD; Royal Philips, Amsterdam, Netherlands) as a part of digital workflow during our routine pathology practice.^23^

**Fig. 1:**
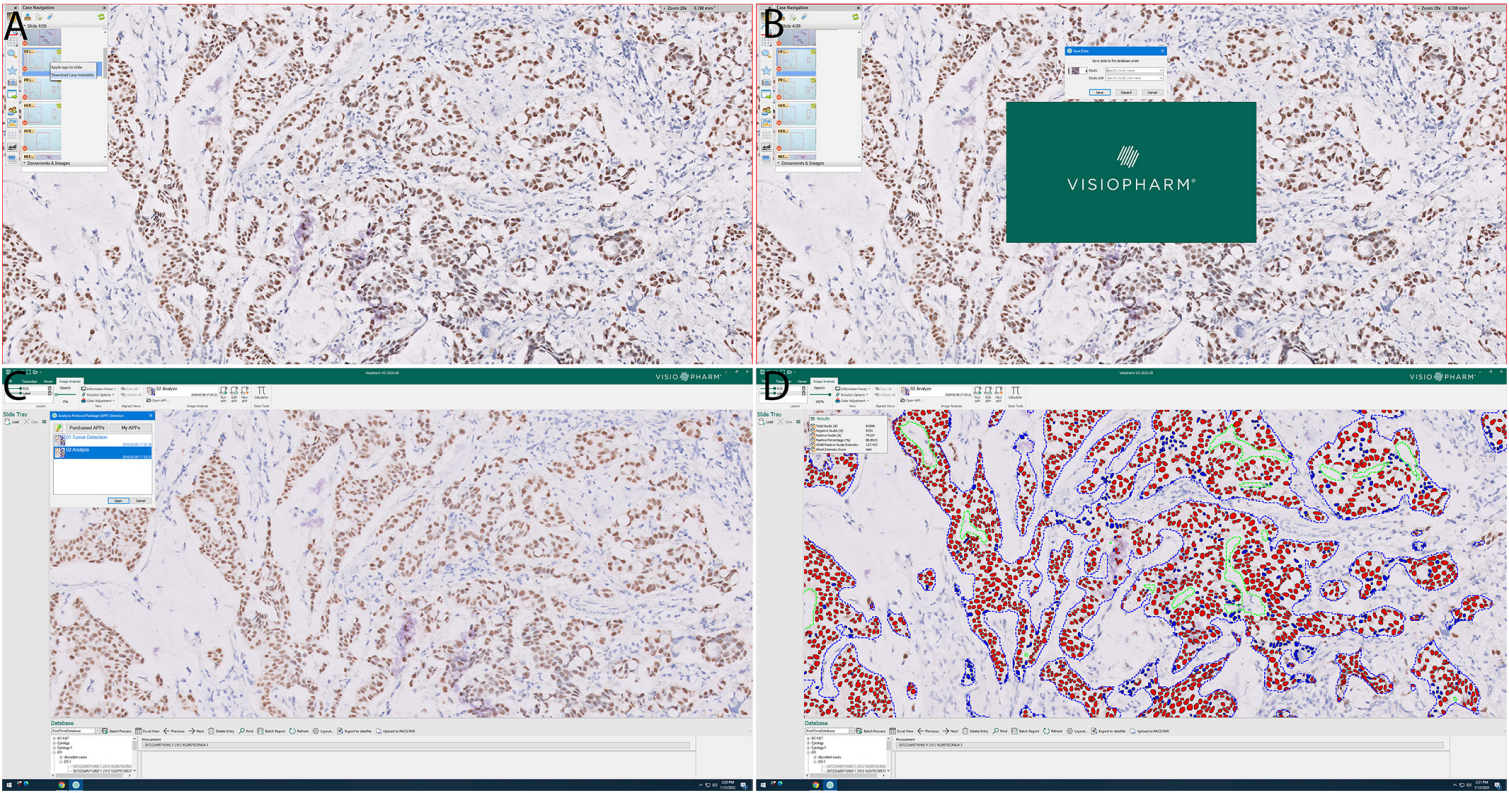
Automated workflow of ER IHC digital imaging analysis (DIA) using Visiopharm coupled with whole slide images (WSI) in the Philips Image Management System (IMS). (A) Downloading case metadata for 1 ER IHC slide in Philips Imaging Managing System. (B) Streaming whole slide image of ER IHC slide by opening its metadata file in Visiopharm. (C) Selecting tumor detection and ER digital imaging analysis algorithms in Visiopharm app center. (D) Analyzing ER results using automated ER digital imaging analsysis algorithm.

**Fig. 2:**
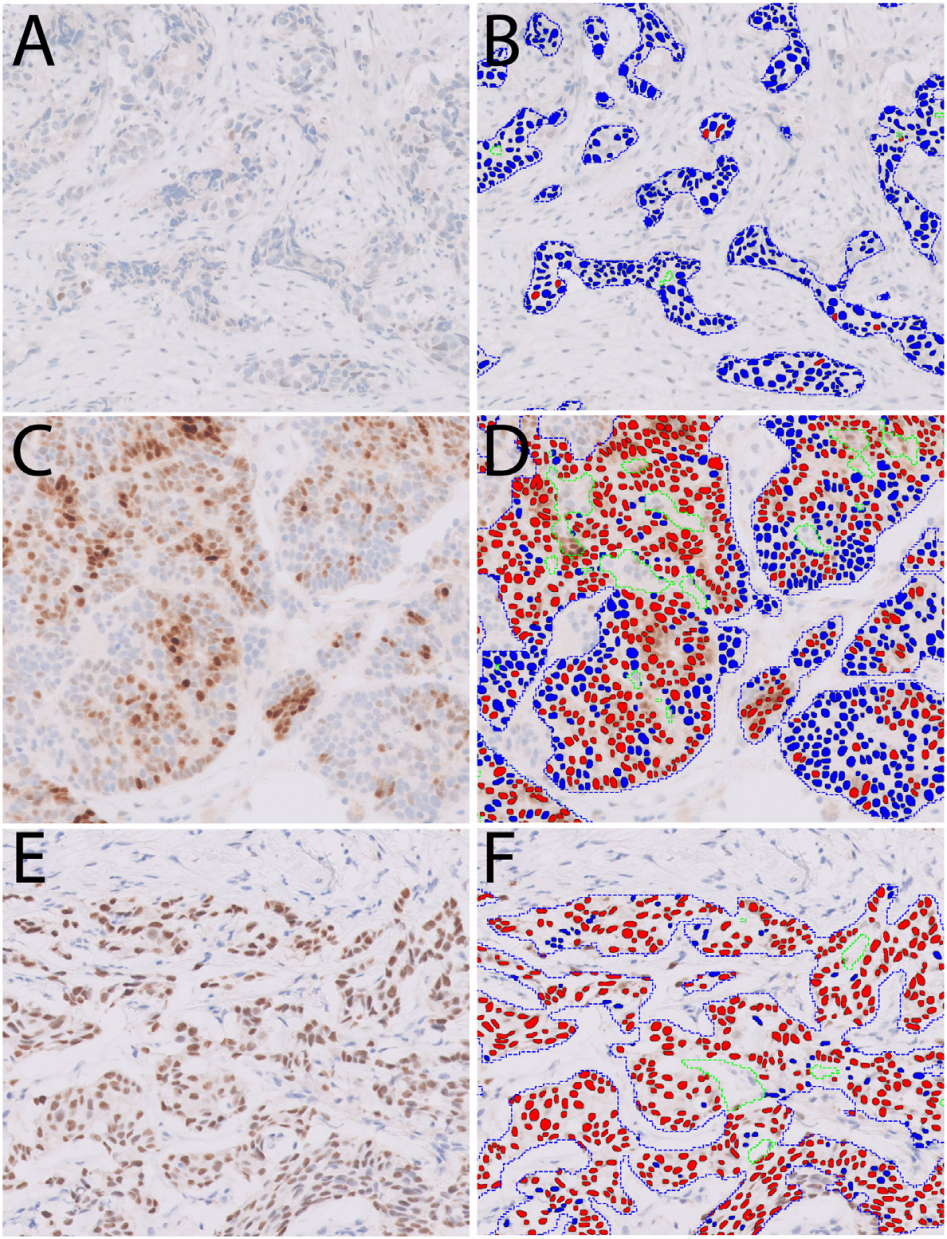
Example of ER quantification by Visiopharm. The left panel of images (A, C, E) shows ER IHC staining, and the right panel (B, D, F) shows cell segmentation and ER quantification with pseudo-colors (blue: negative staining; red: positive staining). Invasive carcinoma is automatically detected and outlined by the algorithm. (A, B) ER with 1.5% positive staining; (C, D) ER with 67% positive staining; (E, F) ER with 96% positive staining. In (D) and (F), red lines outline the tumoral areas, green lines outline the excluded non-tumor components within tumoral areas, such as necrosis, stroma, etc.

Visiopharm (VIS, Visiopharm Integrator System, Hoersholm, Denmark) DIA platform was used to assess the percentage of ER-positive cells on the stained slides. VIS DIA is an automated platform that does not require user supervision. First, ER IHC WSIs were streamed directly from our clinical IMS into VIS platform without downloading/uploading. Second, VIS DIA was able to automatically detect breast carcinoma nuclei using the built-in tumor detection algorithm with robust nuclei detection and segmentation. Finally, VIS DIA analyzed ER IHCs to divide all tumor nuclei into ER negative, weak positive, moderately positive, and strong positive staining based on the DAB intensity after setting the optimal color deconvolution (Fig. 1). The results were exported as an excel file. Some representative images with different ER staining are demonstrated in Fig. 2.

### Statistical analyses

Concordance was measured using the overall percent agreement (OPA) between the VIS DIA reads and pathologists’ reads. OPA was calculated as a ratio of the numbers of cases which DIA’s read was in agreement with original pathologists’ read to the total number of cases. Statistical analysis was performed using SAS version 9.4 for Windows (SAS Institute, Inc, Cary, NC). An adjusted P-value of <0.05 was considered significant.

## Results

### Demographic characteristics of the study cohort

The study cohort was composed of 97 invasive breast carcinomas, including 73 biopsies and 24 resection specimens. There were 56 invasive ductal carcinomas, 3 invasive lobular carcinomas, 2 mixed ductal/lobular carcinomas, and 36 metastatic carcinomas (liver:12, bone:8, axillary lymph nodes:6, brain:5, supraclavicular lymph node: 3, lung:1, chest wall:1). Seventy-three (75.3%) cases were ER-positive, 40 (41.2%) cases were PR-positive, and 16 (16.5%) cases were HER2-positive (Table 1).

**Table 1:** Demographic features of study cohort.

		Cases (n=97)	
		Case #/average	%/range
Age (years)		57	32–93
Specimen	Biopsy	73	75.3%
	Resection	24	24.7%
Histologic type	IDC	56	57.7%
	ILC	3	3.1%
	Mixed IDC/ILC	2	2.1%
	Metastatic carcinoma	36	37.1%
Estrogen receptor	Positive	73	75.3%
	Negative	24	24.7%
Progesterone receptor	Positive	40	41.2%
	Negative	57	58.8%
HER2 IHC	Negative (0/1+)	52	53.6%
	Equivocal (2+)	29	29.9%
	Positive (3+)	16	16.5%

Abbreviations: IDC: invasive ductal carcinoma; ILC: invasive lobular carcinoma, IHC: immunohistochemistry.

### Correlation between ER IHC automated DIA scores and pathologists’ scores

The overall concordance between pathologists’ reads and VIS reads was excellent (93.8%). Out of the 73 ER-positive cases, Visiopharm (VIS) DIA categorized 70 (95.9%) as ER-positive. Twenty-one (87.5%) of the 24 ER-negative cases were also classified as ER-negative by VIS DIA platform (Table 2). Since low ER expressing breast carcinomas (1–10% ER positivity) are usually treated like triple negative breast carcinomas, we further investigated the concordance between pathologists’ reads and VIS reads using a 3-tiered system (ER <1%, ER 1–10%, ER >10%) and the results are shown in Table 3. One case had 85% on original report but 10% by VIS. The cause for this large difference was due to the cutoff threshold in VIS. After adjustment, this case was reported in 70%–80% range by VIS. There were 3 cases with low ER on original reports but more than 10% on VIS. All these 3 cases had DCIS intermixed with invasive carcinoma, causing false increase of positive percentage.

**Table 2:** Comparison between original reads of estrogen receptor immunohistochemistry positivity with digital imaging analysis’ reads and pathologists’ reads.

		Original read				Total	Concordance
		ER-positive		ER-negative			
Total case#		73		24		97	
DIA read	ER-positive	70	95.9%	3	12.5%	73	93.8%
	ER-negative	3	4.1%	21	87.5%	24	
Pathologist read-1	ER-positive	69	94.5%	3	12.5%	72	92.8%
	ER-negative	4	5.5%	21	87.5%	25	
Pathologist read-2	ER-positive	73	100.0%	0	0.0%	73	100.0%
	ER-negative	0	0.0%	24	100.0%	24	

Abbreviations: DIA: digital imaging analysis; ER: estrogen receptor.

**Table 3:** Comparison between original reads and digital imaging analysis’ reads of estrogen receptor immunohistochemistry in 3 categories (ER >10%, ER 1–10%, and ER <1%).

		Original read						Total
		ER >10%		ER 1–10%		ER <1%		
Total		62		11		24		97
VIS read	ER >10%	61	98.4%	3	27.3%	0	0.0%	64
	ER 1–10%	1	1.6%	5	45.5%	3	12.5%	9
	ER <1%	0	0.0%	3	27.3%	21	87.5%	24

**Fig. 3:**
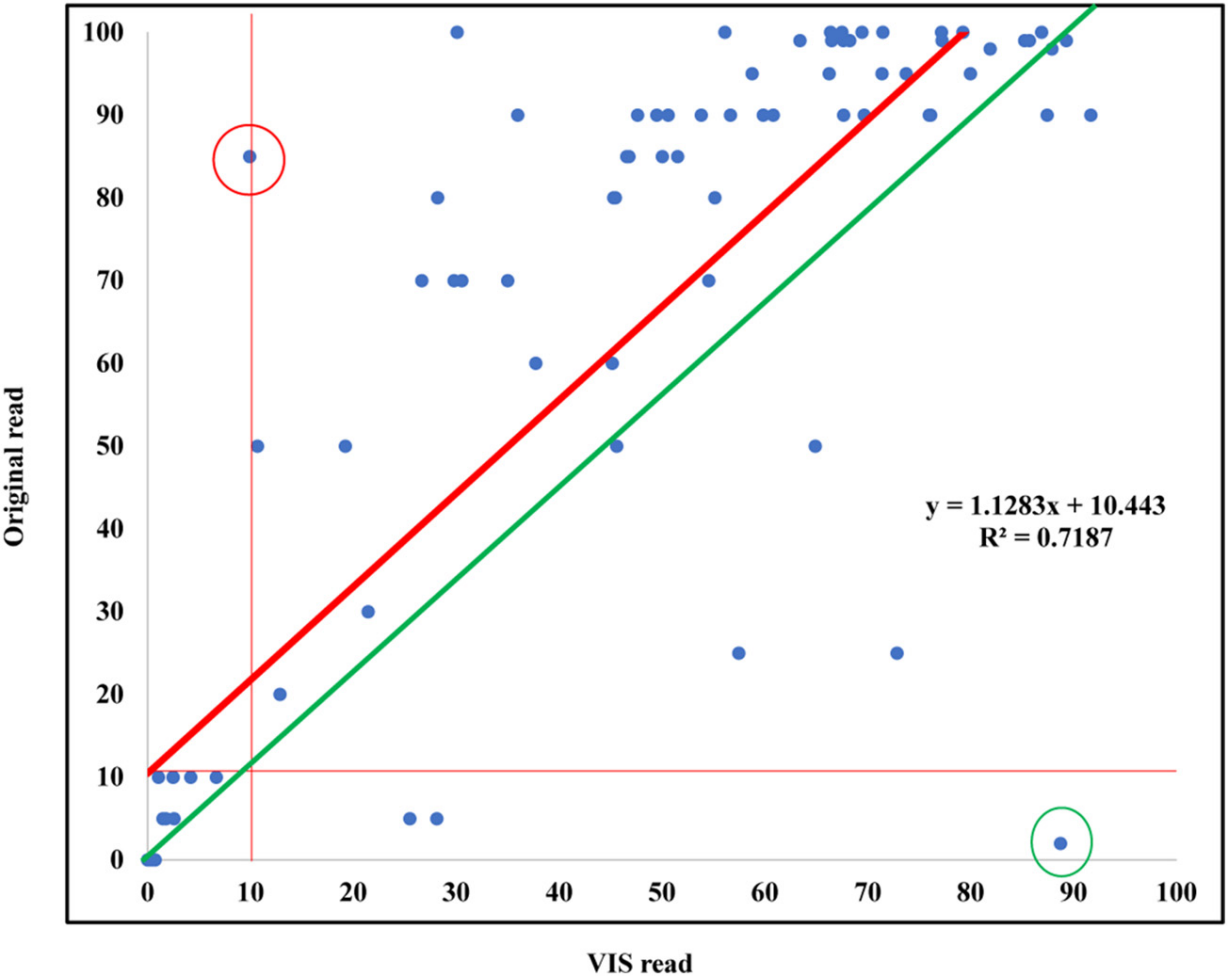
Correlation between the percentage of ER positive nuclei evaluated by DIA and by pathologists. The Pearson correlation coefficient (r) was 0.84776 (n = 97; P < .0001). (y = 1.1283x + 10.443. R^2^ = 0.7187) The red circled case had 85% on original report and 10% by VIS as shown on the histogram and this was not a discordant case but had a large difference for the percentage of ER positively stained tumor cells. The cause for this large difference may be due to the cutoff threshold in VIS. After adjustment, this case was reported in 70%–80% range by VIS. The green circled case in Fig. 3 represents a case with 2% of ER on the original report but 89% by VIS. This large percentage of difference was caused by the presence of DCIS which had more positively stained cells than invasive carcinoma. The other 2 cases with low ER on original reports but more than 10% on VIS also had DCIS intermixed with invasive carcinoma, causing false increase of positive percentage.

Pearson Correlation Coefficient of the percentage of ER positive nuclei between the original reads and VIS reads was 0.84776 (n = 97; P < .0001) [y = 1.1283x + 10.443 (R^2^ = 0.7187)] (Fig. 3). This formula and the diagram demonstrate DIA reads were lower than pathologists’ reads across the board.

In addition, 2 pathologists evaluated ER IHC WSIs independently. The concordance between pathologist 1 and the original read was 92.8% while that between pathologist 2 and original read was 100% (Table 2). Hence, VIS-automated DIA’s performance was comparable with the manual estimation of ER by pathologists.

### Cases with discordant ER results and pitfalls in automated ER IHC DIA

**Fig. 4:**
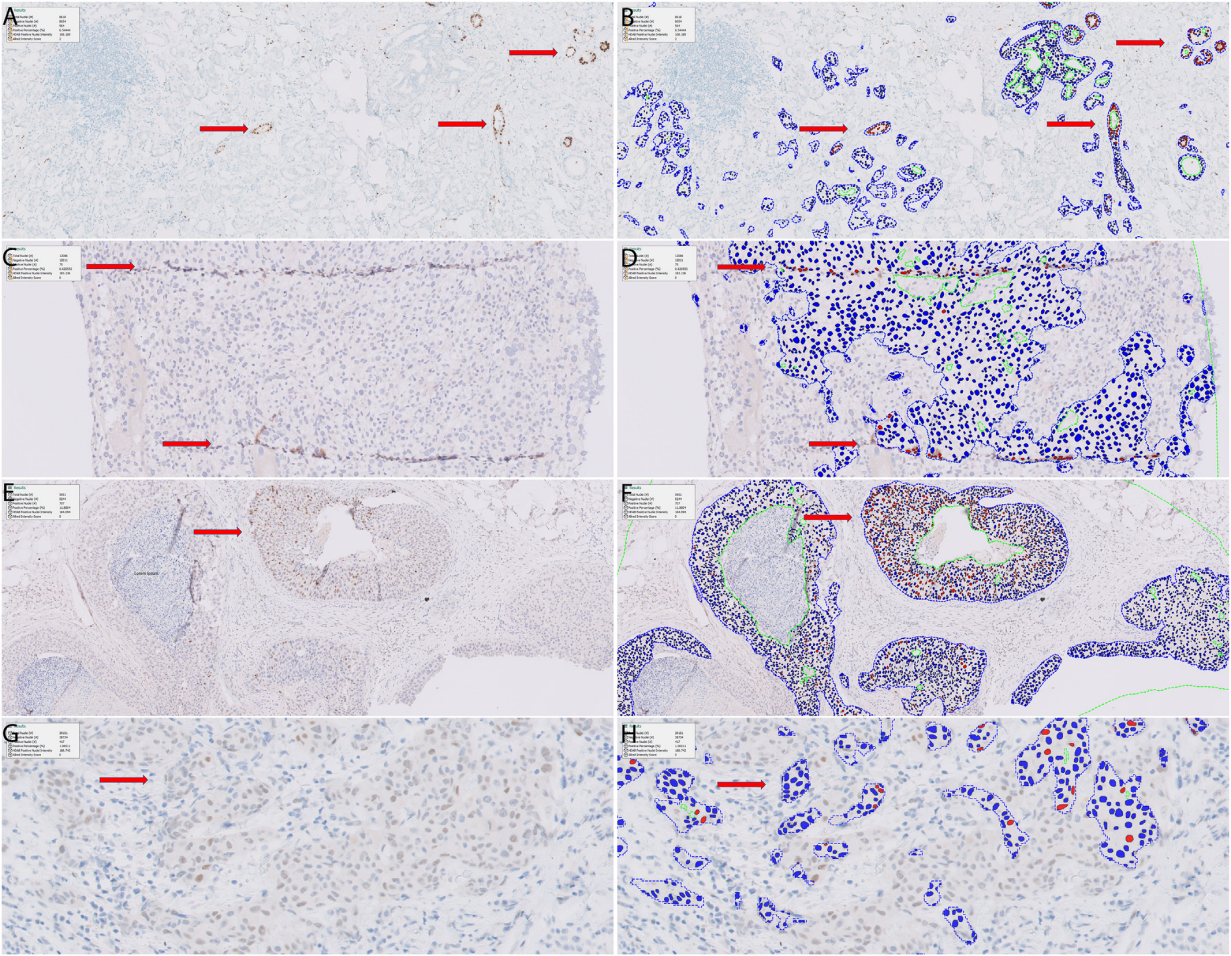
Representative images from cases with discordant results. (A, B) One false-positive case with intermixed benign glands. (C, D) One false-positive case due to tissue folds. (E, F) One false-positive case with staining in DCIS. (G, H) One false-negative due to weak nuclear staining. (A, C, E, G) Original ER-stained immunohistochemical images. (B, D, F, H) Immunohistochemical images with pseudo-colors (blue: negative staining; red: positive staining).

Discordance between pathologists’ read and VIS reads was seen in 6 cases, including 3 ER-negative and 3 ER-positive cases, respectively. The detailed information of these cases is summarized in Table 4. Briefly, all 3 false-negative cases had very weak ER staining and no more than 10% positivity. The causes for false-positive DIA included intermixed benign glands in tumor area (Fig. 4A-B), ductal carcinoma in-situ (DCIS) components (Fig. 4E-F), and tissue folding (Fig. 4C-D). On the other hand, faint ER staining caused false negative DIA results (Fig. 4G-H). After manually removing the false positivity causing areas, DIA was able to analyze those 3 false-positive cases to be negative. After adjustment of the threshold used to separate positive from negatively stained cells, all 3 false-negative cases were re-analyzed by DIA as positive (Table 4).

**Table 4:** Six cases with discordant estrogen receptor results between digital imaging analysis and original reads.

Case #	ER DIA (positive/negative)	Initial ER DIA value (%)	ER DIA value after manual correction (%)	ER original read (positive/negative)	ER original read value (%)	Potential reasons for discordance
1	Positive	8.9%	0.73%	Negative	0.0	Scattered benign glands were included in ROI
2	Positive	15%	0.0%	Negative	0.0	DCIS is included in ROI
3	Positive	1.6%	0.6%	Negative	0.0	Tissue fold resulting in non-specific staining
4	Negative	0.81%	1.5%	Positive	5.0%	Weak staining
5	Negative	0.96%	2.5%	Positive	10.0%	Weak staining
6	Negative	0.41%	1.06%	Positive	10.0%	Weak staining

Abbreviations: DIA: digital imaging analysis; ER: estrogen receptor; ROI: region of interest; DCIS: ductal carcinoma in situ.

## Discussion

Studies have demonstrated that DIA can produce rapid, uniform results with improved precision for biomarker assessment, such as ER.^14^, ^15^, ^16^, ^17^, ^18^, ^19^ Most studies examined algorithms that require input and training by pathologists without automated digital workflow, which required separate slide scanning, WSI uploading to DIA platform, manual selection of region of interest (ROI), causing additional workload and delayed results.^20^^,^^21^

This study aimed to validate automated ER IHC DIA in a real clinical digital workflow and provided critical information highlighting the importance regarding its innovation, automation, accuracy, and the time-consumed. Like other surgical pathology slides, ER IHC slides were scanned and stored in pathology IMS during our routine digital workflow. For automated DIA, ER IHC WSIs were streamed directly from IMS into VIS platform without downloading/uploading. VIS DIA automatically detected breast carcinoma nuclei on ER IHC WSIs using the built-in tumor detection algorithm, divided all tumor nuclei into ER negative and positive staining and calculated the percentage of positively stained tumor nuclei and staining intensity. The entire DIA process was performed by our image analysis specialist, who has no diagnostic pathology expertise. In addition, once all ER IHC WSIs were streamed into VIS platform, a batch DIA process with all WSIs was performed instead of processing WSI one-by-one. This batch process was fully automatic, without requiring any manual intervention. The results for all the cases were exported as an excel file at the end. The time spent on DIA for each case was recorded to be an average of 2.87 min, however, majority of the time was spent on the final imaging analysis. Breast biomarkers are routinely evaluated in batches at many institutions; therefore, this automated batch process will save time and labor.

Our data from the automated DIA in a clinical setting has demonstrated that automated ER IHC DIA is a reliable measurement for ER protein expression, showing an excellent concordance with pathologists’ manual scoring (93.8%). Our results are consistent with previous studies which have shown high agreement between ER DIA and manual scoring in breast cancer specimens.^17^^,^^18^^,^^20^^,^^22^ Pearson correlation analysis between DIA and pathologists’ reads revealed a formula of y = 1.1283x + 10.443 (R^2^ = 0.7187), suggesting DIA generally yielded lower values than pathologists. This may be caused by overestimation from pathologists or a higher threshold for separating positive from negatively stained cells in the DIA algorithm. The latter possibility is more likely since the 3 false-negative cases were correctly assessed after adjusting the threshold.

It is potentially feasible to apply DIA on cytology specimens such as cell block sections. Previous study has demonstrated non-inferiority for interpreting breast cancer biomarkers on cell block WSIs.^24^ Our preliminary data suggest tumor detection algorithm in current ER DIA can reliably detect tumor cells in cell block sections. In addition, breast carcinomas in cytology specimens are mostly metastasis and in situ carcinoma component does not exist to interfere the interpretation of ER IHCs. Additionally, we have tested the ER DIA for progesterone receptor (PR) IHC quantification and obtained good concordance results. It would be interesting to see how this DIA works for other markers with nuclear stain.

We have identified several pitfalls in the automated ER DIA process, including intermixed benign ducts, DCIS components, and tissue folding, that cause false-positive results; and very faint ER IHC staining to cause false-negative results. These pitfalls are not infrequent (6/97, 6%), but most of them can be avoided by simple manual annotation of region-of-interest (i.e., excluding intermixed benign ducts, DCIS components, and tissue folding) or by adjustment of the threshold used to separate positive from negatively stained cells.

To summarize, we demonstrate that automated ER IHC DIA is a valid tool to determine ER status in breast carcinoma with a high concordance to pathologists’ scoring. Furthermore, we show that integrating automated biomarker DIA into a busy clinical digital workflow is feasible and may save time and labor for pathologists.

## Declaration of interests

The authors declare that they have no known competing financial interests or personal relationships that could have appeared to influence the work reported in this paper.
